# Finite Mixture Models Based on Pain Intensity, Functional Disability and Psychological Distress Composite Assessment Allow Identification of Two Distinct Classes of Persistent Spinal Pain Syndrome after Surgery Patients Related to Their Quality of Life

**DOI:** 10.3390/jcm10204676

**Published:** 2021-10-13

**Authors:** Amine Ounajim, Maxime Billot, Pierre-Yves Louis, Yousri Slaoui, Denis Frasca, Lisa Goudman, Manuel Roulaud, Nicolas Naiditch, Bertille Lorgeoux, Sandrine Baron, Kevin Nivole, Mathilde Many, Nihel Adjali, Philippe Page, Bénédicte Bouche, Elodie Charrier, Laure Poupin, Delphine Rannou, Géraldine Brumauld de Montgazon, Brigitte Roy-Moreau, Chantal Wood, Raphaël Rigoard, Romain David, Maarten Moens, Philippe Rigoard

**Affiliations:** 1 PRISMATICS Lab (Predictive Research in Spine/Neuromodulation Management and Thoracic Innovation/Cardiac Surgery), Poitiers University Hospital, 86021 Poitiers, France; Maxime.BILLOT@chu-poitiers.fr (M.B.); Manuel.ROULAUD@chu-poitiers.fr (M.R.); nicolas.naiditch@gmail.com (N.N.); Bertille.LORGEOUX@chu-poitiers.fr (B.L.); sandrine.baron@chu-poitiers.fr (S.B.); Kevin.NIVOLE@chu-poitiers.fr (K.N.); mathilde.many@chu-poitiers.fr (M.M.); Nihel.adjali@chu-poitiers.fr (N.A.); dr.bouche@gmail.com (B.B.); chantalwood@orange.fr (C.W.); romain-david@hotmail.fr (R.D.); Philippe.RIGOARD@chu-poitiers.fr (P.R.); 2 Laboratoire de Mathématiques et Applications UMR 7348, CNRS, University of Poitiers, 86073 Poitiers, France; yousri.slaoui@math.univ-poitiers.fr; 3 AgroSup Dijon, PAM UMR 02.102, Université Bourgogne Franche-Comté, 21000 Dijon, France; pierre-yves.louis@agrosupdijon.fr; 4 Institut de Mathématiques de Bourgogne, UMR 5584 CNRS, Université Bourgogne Franche-Comté, 21000 Dijon, France; 5 Department of Anaesthesiology and Critical Care, Poitiers University Hospital, 86021 Poitiers, France; denis.frasca@chu-poitiers.fr; 6 INSERM UMR-1246, Universities of Nantes and Tours, 37044 Tours, France; 7 Department of Neurosurgery, Universitair Ziekenhuis Brussel, 1090 Brussels, Belgium; lisa.goudman@gmail.com (L.G.); mtmoens@gmail.com (M.M.); 8 STUMULUS Research Group, Vrije Universiteit Brussel, 1090 Brussels, Belgium; 9 Dyname, UMR 7367, Faculty of Social Sciences, University of Strasbourg, 67083 Strasbourg, France; 10 Department of Spine Surgery & Neuromodulation, Poitiers University Hospital, 86021 Poitiers, France; Philippe.PAGE@chu-poitiers.fr; 11 Pain Evaluation and Treatment Centre, Poitiers University Hospital, 86021 Poitiers, France; Elodie.CHARRIER@chu-poitiers.fr (E.C.); laure.poupin@chu-poitiers.fr (L.P.); delphine.rannou@chu-poitiers.fr (D.R.); 12 Pain Evaluation and Treatment Centre, La Rochelle Hospital, 17000 La Rochelle, France; Geraldine.DEMONTGAZON@ght-atlantique17.fr; 13 Pain Evaluation and Treatment Centre, Nord Deux-Sèvres Hospital, 79000 Niort, France; Roy-Moreau.Brigitte@chnds.fr; 14 CEA Cadarache, Département de Support Technique et Gestion, Service des Technologies de l’Information et de la Communication, 13108 Saint-Paul-Lez-Durance, France; cydran@gmail.com; 15 Physical and Rehabilitation Medicine Unit, Poitiers University Hospital, University of Poitiers, 86021 Poitiers, France; 16 Prime Institute UPR 3346, CNRS, ISAE-ENSMA, University of Poitiers, 86360 Chasseneuil-du-Poitou, France

**Keywords:** PSPS, FBSS, chronic pain, health-related quality of life, mixture models analysis, personalized pain management, chronic pain after spinal surgery

## Abstract

Persistent Spinal Pain Syndrome Type 2 (PSPS-T2), (Failed Back Surgery Syndrome), dramatically impacts on patient quality of life, as evidenced by Health-Related Quality of Life (HRQoL) assessment tools. However, the importance of functioning, pain perception and psychological status in HRQoL can substantially vary between subjects. Our goal was to extract patient profiles based on HRQoL dimensions in a sample of PSPS-T2 patients and to identify factors associated with these profiles. Two classes were clearly identified using a mixture of mixed effect models from a clinical data set of 200 patients enrolled in “PREDIBACK”, a multicenter observational prospective study including PSPS-T2 patients with one-year follow-up. We observed that HRQoL was more impacted by functional disability for first class patients (*n* = 136), and by pain perception for second class patients (*n* = 62). Males that perceive their work as physical were more impacted by disability than pain intensity. Lower education level, lack of adaptive coping strategies and higher pain intensity were significantly associated with HRQoL being more impacted by pain perception. The identification of such classes allows for a better understanding of HRQoL dimensions and opens the gate towards optimized health-related quality of life evaluation and personalized pain management.

## 1. Introduction

Persistent Spinal Pain Syndrome Type 2 (PSPS-T2) [1], also known as Failed Back Surgery Syndrome, is characterized by post-operative chronic pain, following one or several spinal surgical procedure(s) and persisting beyond the healing process [1]. With a prevalence of approximately 20% of patients who have undergone spine surgery, PSPS-T2 represents a major public health problem and a financial burden [2]. Besides pain perception, PSPS-T2 patients present major psychological distress and functional disability, which lead to an overall decrease in Health-Related Quality of Life (HRQoL) [3,4,5]. It remains difficult to accurately measure the impact of pain perception, psychological distress and functional disability on HRQoL, which could accurately characterize the PSPS-T2 patient population, a key objective being to optimize their pain management.

Previous research, investigating the relationship between quality of life and clinical outcomes in various pathologies, showed that pain intensity, psychological distress and functional disability were all correlated with HRQoL [6,7,8,9]. More specifically, Kovacs et al. [10] investigated the correlation between pain intensity, functional disability and HRQoL in a primary care setting in 195 patients suffering from low back pain. The authors reported that HRQoL had a significant negative correlation with pain intensity and functional disability (coefficient of correlation r ranging from −0.347 to −0.672). Furthermore, in a cross-sectional study on 200 patients with general chronic pain, Rapti et al. [11] found significant negative correlations between HRQoL and pain intensity (r = −0.65), and depression score (r = −0.63). However, these results were all obtained from an overall correlation, which made their authors unable to identify potential specific subpopulations presenting different profiles related to the variable impact of pain intensity, functional disability, and psychological distress on HRQoL. We hypothesized that the clustering of patients based on the impact of these three chronic pain components on HRQoL could be performed so as to identify and to characterize specific PSPS-T2 patient classes. This might finally impact on patient medical management.

The identification of clusters can be performed using finite mixture models [12,13,14]. These models provide two or more distinct homogeneous latent classes of individuals based on the associations between different variables. For example, Chen et al. [13] used finite mixture analysis to extract the trajectories of pain over a five-year follow-up period in patients with low back pain. The authors isolated four distinct pain trajectory classes: no or occasional mild pain, persistent mild pain, fluctuating pain, and persistent severe pain. By characterizing this population, they reported that level of education (<16 years), restricted work or unemployment, pain intensity, disability, pain duration (>3 years), catastrophizing, anxiety and depression were associated with pain trajectories. While the clusters were based on trajectories, their study was not intended to determine clusters according to how chronic pain components (i.e., pain intensity, functional disability and psychological distress) could be related to HRQoL. Therefore, clustering and characterization of chronic low back pain patients regarding association between different pain components have yet to be attempted, especially in the PSPS-T2 population.

The main goal of this real-life prospective multicenter study was to identify, after 12-months of follow-up, different population subgroups based on the specific impact of pain intensity, functional disability and psychological distress on HRQoL in 200 PSPS-T2 refractory patients. Our secondary objective was to characterize these subgroups using clinical, psychological and social factors to refine the model of personalized medical management.

## 2. Materials and Methods

### 2.1. Data Collection and PREDIBACK Study Description

The data used for the purpose of this research consisted of PSPS-T2 patients included in the prospective, multicenter, observational PREDIBACK study. The primary objective of the PREDIBACK study was to characterize PSPS-T2 patients based on clinical, psychological and sociodemographic factors. Two hundred patients were consecutively recruited in the pain management department of 5 French centers (Angoulême, Bressuire, La Rochelle, Niort and Poitiers) and monitored at baseline, 3-month, 6-month, 9-month and 12-month follow-ups. Patient recruitment started in January 2017 and was completed in March 2018. The study was approved by the ANSM (2016-A01144-47) and the Ethics Committee (CPP Ouest III) and declared at https://clinicaltrials.gov/ct2/show/NCT02964130 (accessed on 15 March 2021).

### 2.2. Patient Selection

#### 2.2.1. Inclusion Criteria

PSPS-T2 patients were identified at each site through standard clinical practice. To be eligible for this study, patients had to have had at least one spinal surgery, post-operative leg and/or low back pain for at least six months, and an average global pain score greater than or equal to 4 as measured by the Numeric Pain Rating Scale (NPRS). Patients at baseline were asked to report their average pain over a week on an NPRS scale (i.e., “Please rate your pain in this scale that best describes your pain on average over the last week”). All the patients gave their informed consent before enrolment.

#### 2.2.2. Non-Inclusion Criteria

Patients with one or several of the following criteria were excluded from the study: patient is or has been treated with spinal cord, subcutaneous or peripheral nerve stimulation or an intrathecal drug delivery system; has life expectancy of less than 12 months beyond study enrollment; patient is unable to undergo study assessments or to complete questionnaires independently; is a member of a vulnerable population; and investigator suspects substance abuse which might confound the study results.

### 2.3. Demographic Variables Encoding

Patient professional activity was encoded as follows: patients were considered as (i) active when they were involved in professional activity at baseline assessment, (ii) inactive when they were retired, without any professional activity, or on sick leave, disability and long-term sick leave. There were no patients that were still students. Two patients were housewives and were considered as active. Furthermore, to identify active patients with arduous working conditions, they were asked to characterize their work as “physically demanding” or not.

### 2.4. Clinical Outcome Measurements

#### 2.4.1. Health-Related Quality of Life

HRQoL was assessed by the EuroQol 5 Dimensions 5-Level questionnaire (EQ-5D-5L) [15]. The questionnaire comprises 5 items including pain intensity, mobility, self-care, daily activities and psychological state (anxiety or depression). Each item consists of a 5-level Likert scale ranging from “I have no problem” to “I am unable to”. The maximum score of 1 indicates the best possible HRQoL. EQ-5D has demonstrated moderate to excellent validity and reliability in a large number of studies of chronic conditions [16,17,18]. The main outcome of this study was the EQ-5D index and not the EQ-5D VAS.

#### 2.4.2. Pain Intensity

Pain intensity was assessed using the NPRS [19] which is an 11-point (0–10) unidimensional scale where the patient selects the number that best represents his overall pain intensity during the last week. The 11-point numeric scale ranges from 0 representing “no pain” to 10 representing “the worst pain imaginable”.

#### 2.4.3. Functional Disability

The Oswestry Disability Index (ODI) questionnaire was used to assess functional disability [20]. The ODI indicates the degree of disability induced by low back pain including pain intensity, degree of disability for personal care, lifting, walking, sitting, standing, sleeping, sexual life, social life and travelling. The questionnaire consists of 10 items ranging from 0 (high ability to perform the task) to 5 (inability to perform the task). The score is expressed as a percentage of disability (sum of item scores divided by 50) where a low percentage represents minimal disability and a high percentage represents severe disability to being bed-bound. The ODI is one of the questionnaires which has been validated and showed internal consistency ranging from moderate to excellent [21,22].

#### 2.4.4. Psychological Distress: Anxiety/Depression and Coping Strategies

The Hospital Anxiety and Depression Scale (HADS) [23] was used to investigate anxiety and depression symptoms and their severity. The questionnaire consists of 14 items, each of them comprising 4 levels and representing either symptoms of anxiety or depression. The total score ranges from 0 to 24 for each category (depression or anxiety). A score of 11 or above indicates a definite symptomatology. The HADS questionnaire shows high internal consistency in the acute low back pain population and in the general population [24,25]. HADS has been used as an assessment tool in several studies on chronic back and leg pain [26].

The French adaptation of the Coping Strategies Questionnaire (CSQ) [27] was used to assess patients’ self-rated use of cognitive and behavioral strategies to cope with pain. It consists of 6 subscales for cognitive strategies including pain ignorance (5 items), reinterpretation (4 items), diversion (5 items), self-encouragement (4 items), catastrophizing (6 items) and praying/hoping (3 items). Each item is a Likert scale ranging from 1 “never” to 4 “always” indicating how frequently the strategy is used to cope with pain. The praying subscale was not used in this study due to an ethics committee’s request.

### 2.5. Statistical Analysis

Statistical analyses were conducted using the R software (Version 3.6.0, R Foundation for Statistical Computing, Vienna, Austria).

Variables were described by their means (standard deviation) or by their number (percentage) depending on whether the variable was qualitative or quantitative.

Missing values were not imputed, and data were analyzed according to an available-case principle. All tests were two-tailed and *p*-values < 0.05 were considered statistically significant.

#### 2.5.1. Measurements Internal Consistency

In order to assess the internal consistency of HADS, EQ-5D, and ODI questionnaires for PSPS-T2 population, Cronbach’s alpha and its confidence interval were calculated for each measurement questionnaire. Internal consistency measures whether several items from the same questionnaire measure the same general construct based on the amount of shared information. Internal validity of these questionnaires has never been studied for the PSPS-T2 population. Confidence intervals were estimated using the Bootstrap technique [28]. A Cronbach alpha between 0.6 and 0.7 is considered acceptable; a Cronbach alpha between 0.7 and 0.8 is considered good; and a Cronbach alpha greater than 0.8 is considered excellent.

#### 2.5.2. Correlation between EQ-5D, NPRS, ODI and HADS Depression Scores

In order to estimate the correlations between our measurement variables, Pearson’s simple correlation was calculated between the different variable assessments at baseline and 12-month follow-up. Baseline and 12-month follow-up correlations were compared using a z-test after conducting Fisher transformation on the coefficients.

#### 2.5.3. Clustering of the Impact of Pain Intensity, Functional Disability and Depression on HRQoL

Mixture of mixed effect models [29] were used in order to extract the latent classes based on the effects of functional disability (ODI score), pain intensity (NPRS) and depression (HADS depression score) on HRQoL (EQ-5D index). The optimal number of latent classes was identified using the Bayesian Information Criterion (BIC) [30]. We estimated the model for different numbers of classes (ranging from 1 to 4 classes) and the model with the smallest BIC was considered as the final one. The intercept was fixed between the classes. In the mixed effects model of the mixture, the explained variable was HRQoL measured by the EQ-5D index at baseline, 3, 6, 9 and 12-month follow-up. The explanatory variables were global NPRS, ODI score and HADS depression score also measured at baseline, 3, 6, 9 and 12-month follow-up. We included a random intercept effect to account for within-patient mean differences. Standardized coefficients and their 95% confidence intervals were reported.

#### 2.5.4. Baseline Characteristics Influencing Class Membership

After the classes and their respected effects were estimated, they were used as a binary dependent variable (the optimal number of extracted classes was 2) to determine the impact of sociodemographic, psychological and cognitive-behavioral variables on class membership. Normality of distributions was verified using the Shapiro–Wilk test. Relationship between qualitative variables and class membership was verified using the Fisher exact test for non-ordinal qualitative variables and the Cochran–Armitage test for ordinal variables. The Wilcoxon rank-sum test was used to test the relationship between class membership and quantitative variables. NPRS, ODI percentage, HAD depression and anxiety scales, gender, level of education, CSQ catastrophizing, ignorance (ignoring pain sensation), reinterpretation (reinterpreting pain sensations), diversion (diverting attention from pain) and self-encouragement subscales, perceived arduous working conditions, and the interaction between gender and perceived arduous working conditions at baseline were included simultaneously in a logistic regression model in order to assess the adjusted effects of these variables on class membership (i.e., estimate the effect of each variable while taking into account other confounding variables). Model coefficients and their 95% confidence intervals were reported.

We also studied the relationship between level of education, perceived arduous working conditions and coping strategy scores (Pearson correlation coefficients), in order to help us to interpret our bivariate and multiple regression results.

## 3. Results

### 3.1. Study Population

Out of the 200 patients enrolled, 168 (84%) completed data at 3-month, 166 (83%) at 6-month, 145 (72.5%) at 9-month, and 146 (73%) at 12-month follow-up. Two patients completed only the sociodemographic and clinical data, but did not complete any of the evaluation questionnaires; therefore, they were used only for descriptive analysis and were removed from the other analyses. The relatively high percentage of dropout was due to the fact that the study was observational and the treatment the patients receive does not change whether they participated or not in the study.

### 3.2. Measurement Internal Consistency

The assessment tools used in this study have shown acceptable to good internal consistency in our dataset with a Cronbach alpha of 0.693 (CI95% = [0.605, 0.758]) for EQ-5D, 0.801 (CI95% = [0.743, 0.848]) for the ODI, and 0.775 (CI95% = [0.716, 0.814]) for the HADS scores.

### 3.3. Baseline Characteristics and Outcomes

Table 1 presents the demographic and clinical characteristics of the study population at baseline. The study participants mean age was 53 ± 13 years and 111 (56.1%) were females. Ninety-nine patients (50.5%) had at least two spinal surgeries, 59 patients (29.8%) had at least three spinal surgeries, and the 40 remaining patients (20.2%) had four or more spinal surgeries. At baseline, the study sample had a mean EQ-5D score of 0.27 ± 0.24, a mean global NPRS of 6.1 ± 1.5, an ODI percentage of 34.3 ± 11.7%, and a mean HAD depression score of 8.6 ± 3.9.

### 3.4. Correlation between EQ-5D and ODI, NPRS and HADS Depression

Correlations between different scores are presented in Table 2.

The EQ-5D index was significantly correlated at baseline and 12-month follow-up with NPRS (r_baseline_ = −0.35 and r_12-month_ = −0.55, *p* < 0.0001), ODI score (r_baseline_ = −0.66 and r_12-month_ = −0.77, *p* < 0.0001) and HADS depression score (r_baseline_ = −0.44 and r_12-month_ = −0.56, *p* < 0.0001). ODI was moderately correlated with HADS depression score (r_baseline_ = 0.40 and r_12-month_ = 0.57, *p* < 0.0001) and NPRS score (r_baseline_ = 0.44 and r_12-month_ = 0.54, *p* < 0.0001). NPRS score was moderately correlated with HADS depression score (r_baseline_ = 0.33 and r_12-month_ = 0.39, *p* < 0.0001). The correlation coefficients between EQ-5D index and NPRS score (*p* = 0.011), and ODI score (*p* = 0.019) significantly increased from baseline to the 12-month follow-up (Table 2). The absolute difference between baseline and 12 months of EQ-5D index was also correlated with the absolute difference in ODI score (r = −0.63, *p* < 0.0001), the absolute difference in NPRS (r = −0.41, *p* < 0.0001), and the absolute difference in depression HADS score (r = −0.57, *p* < 0.0001).

### 3.5. Standard 1-Class Mixed Effects Model Results

Table 3 presents the standardized coefficients and standard errors of the effects of NPRS score, ODI score and HADS depression score on EQ-5D index. The standard 1-class mixed effects model showed that NPRS, ODI and HADS depression scores had statistically significant effects on EQ-5D index (*p* < 0.0001). The ODI score had a greater impact on EQ-5D index (standardized coefficient = −0.48, CI95% = [−0.549, −0.411], *p* < 0.0001) compared to the NPRS score (standardized coefficient = −0.13, CI95% = [−0.184, −0.076], *p* < 0.0001) and the HADS depression score (standardized coefficient = −0.20, CI95% = [−0.261, −0.139], *p* < 0.0001). The fixed effects of the NPRS and HADS scores and the ODI percentage explained 54% of the total EQ-5D index variance, while the entire model including both fixed and random effects explained 77% of the total EQ-5D index variance.

### 3.6. Two-Class Mixed Model Results

**Figure 1:**
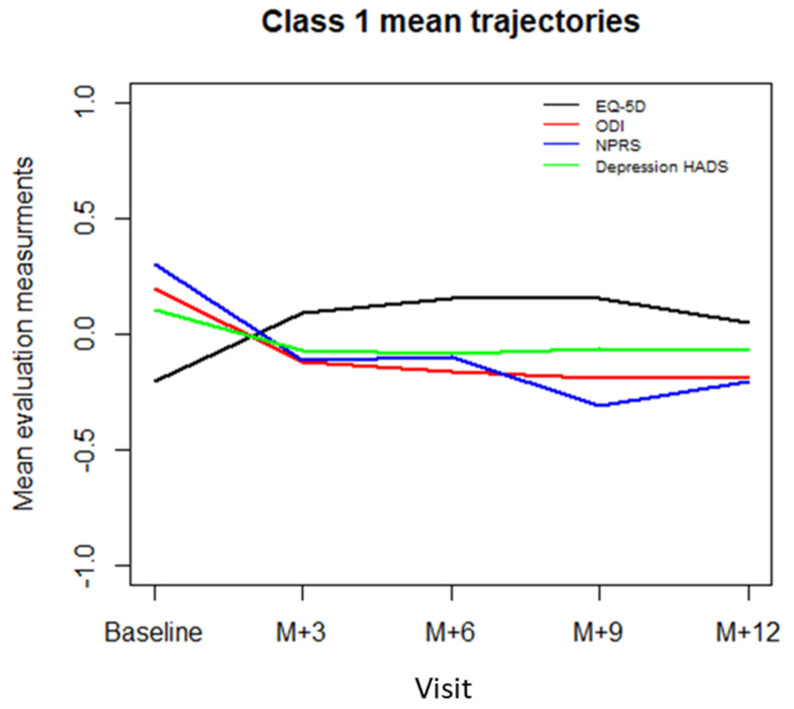
The “functional disability” class 1 mean trajectories for each evaluation criterion at baseline and at 3 (M + 3), 6 (M + 6), 9 (M + 9) and 12 (M + 12) months. EQ-5D: EuroQol-5 Dimensions, ODI: Oswestry Disability Index, NPRS: Numeric Pain Rating Scale, HAD: Hospital Anxiety and Depression scale (depression score).

**Figure 2:**
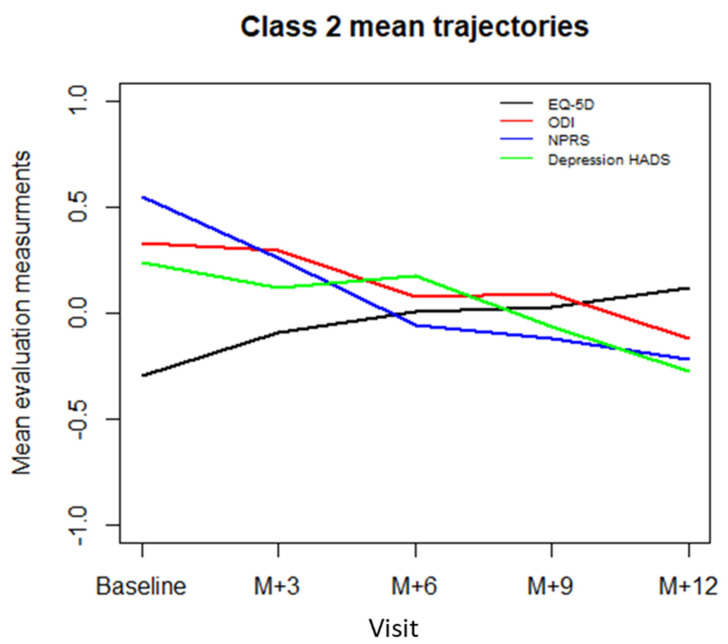
The “pain intensity” class 2 mean trajectories for each evaluation criterion at baseline and at 3 (M + 3), 6 (M + 6), 9 (M + 9) and 12 (M + 12) months. EQ-5D: EuroQol-5 Dimensions, ODI: Oswestry Disability Index, NPRS: Numeric Pain Rating Scale, HAD: Hospital Anxiety and Depression scale (depression score).

Table 3 presents the results of the standard mixed effects model and the mixture of mixed effects models. The mixture of mixed effects models allowed us to extract two latent classes based on the BIC (BIC = 1326.4 for 1-class model, BIC = 1312.3 for 2-class model, BIC = 1319.2 for 3-class model, and BIC = 1334.2 for 4-class model). The posterior mean probability of the first class was 0.75 and the mean posterior probability of the second class was 0.76, indicating an acceptable dissociation between the classes. Mean trajectories of standardized EQ-5D index, NPRS, ODI, and depression HADS scores for class 1 and 2 patients are represented in Figure 1 and Figure 2.

For class 1 patients, who comprised 68.7% (136/198) of the sample patients, the effects on EQ-5D index were statistically significant for the ODI score (standardized coefficient = −0.76, CI95% = [−0.91, −0.61], *p* < 0.0001) and depression HADS score (standardized coefficient = −0.19, CI95% = [−0.28, −0.10], *p* < 0.0001), whereas the effect was not significant for NPRS (standardized coefficient = 0.039, CI95% = [−0.04, 0.12], *p* = 0.46). Based on these results, the class 1 will be referred to as “disability class”.

For class 2 patients, who comprised 31.3% (62/198) of the study sample, the NPRS score (standardized coefficient = −0.35, CI95% = [−0.481, −0.219], *p* < 0.0001) and depression HADS score (standardized coefficient = −0.22, CI95% = [−0.34, −0.11], *p* = 0.0001) had a significant effect on EQ-5D index, whereas the ODI score did not (standardized coefficient = −0.11, CI95% = [−0.30, 0.08], *p* = 0.23). Based on these results, the class 2 will be referred to as “pain intensity class”.

### 3.7. Relationship between Baseline Characteristics and Classes

Bivariate and multiple regression analyses results are presented in Table 4. The bivariate analysis showed that patients in the “disability class” had a higher educational level than patients in the “pain intensity class” (11.36 ± 3.14 years vs. 9.47 ± 4.36 years, CI95% of the difference = [0.63, 3.14], *p* = 0.004). Furthermore, the NPRS score at baseline was lower in the “disability class” than in the “pain intensity class” (5.91 ± 1.41 vs. 6.47 ± 1.57, CI95% of the difference = [0.63, 3.14], *p*-values = 0.02). Baseline HADS anxiety score was also significantly associated with class membership showing that patients in the “disability class” presented higher anxiety levels at baseline than patients in the “pain intensity class” (10.43 ± 3.79 vs. 9.03 ± 3.83, CI95% of the difference = [0.22, 2.57], *p*-values = 0.02). The mean CSQ diversion score was significantly higher in the “disability class” than in the “pain intensity class” (12.68 ± 3.50 vs. 11.30 ± 3.72, CI95% of the difference = [0.22, 2.54], *p* = 0.02). Similarly, higher CSQ pain reinterpretation scores were observed in the “disability class” compared to the “pain intensity class” (6.03 ± 2.55 vs. 5.09 ± 1.71, CI95% of the difference = [0.27, 1.59], *p* = 0.02). Gender, age, pain duration, perceived arduous working conditions, ODI percentage, depression HADS score and CSQ catastrophizing, ignorance and self-encouragement scores had no significant impact on class membership in the univariate analysis (*p* > 0.22).

The multiple logistic regression analysis showed that patients with higher NPRS scores were more likely to belong to the “pain intensity class” compared to patients with lower NPRS (standardized coefficient = 0.65, CI95% = [0.05, 1.24], *p* = 0.029). On the other hand, ODI score and pain duration had no significant impact on class membership (*p* = 0.45 for ODI and *p* = 0.37 for pain duration).

Regarding sociodemographic factors, patients in the “disability class” had higher mean educational level compared to the “pain intensity class” (standardized coefficient = −0.61, CI95% = [−1.15, −0.199], *p* = 0.02). Neither age (*p* = 0.31), nor gender (*p* = 0.32), nor perceived arduous working conditions (*p* > 0.7) had a significant effect on class membership. However, the interaction between gender and perceived arduous working conditions had a statistically significant effect on class membership, showing that men who perceive their job as physical were more likely to belong to the “disability class” than to the “pain intensity class” (standardized coefficient = −0.55, CI95% = [−1.08, −0.03], *p* = 0.027).

Regarding psychological factors and coping strategies, patients with higher HADS depression scores were more likely to belong to the “pain intensity class” (standardized coefficient = 0.88, CI95% = [0.17, 1.59], *p* = 0.012), and patients with higher HADS anxiety scores were more likely to belong to the “disability class” (standardized coefficient = −0.98, CI95% = [−1.70, −0.27], *p* = 0.005). We also found that the HRQoL of patients with higher self-encouragement coping scores were more likely to be in the “pain intensity class” than in the “disability” class (standardized coefficient = 0.81, CI95% = [0.29, 1.34], *p* = 0.011). The remaining coping strategies were not significantly associated with class membership in multiple regression analysis (*p* = 0.07 for catastrophizing, *p* = 0.09 for diversion, *p* = 0.13 for pain ignorance, and *p* = 0.12 for pain reinterpretation).

### 3.8. The Relationship between Level of Education, Perceived Arduous Working Conditions and Coping Strategies

The relationships between the different factors used in the multiple regression analysis showed that level of education was not significantly associated with perceived arduous working conditions (*p* = 0.71) with an average of 10.91 ± 3.10 years of education in non-working patients (including retired patients), 10.42 years ± 4.67 in working patients who did not perceive their job as physical, and 10.92 ± 3.75 years in working patients who perceived their job as physical. Furthermore, statistical analysis did not show significant correlations between educational level and the different CSQ scores for catastrophizing (r = −0.13, *p* = 0.09), pain ignorance (r = −0.10, *p* = 0.19), pain reinterpretation (r = 0.04, *p* = 0.62), diversion (r = 0.05, *p* = 0.49) and self-encouragement (r = 0.13, *p* = 0.09) scores. We also observed moderate correlations between HADS anxiety score and CSQ catastrophizing score (r = 0.57, *p* < 0.0001) and between HADS anxiety score and CSQ ignorance score (r = −0.29, *p* = 0.0001). No significant correlation was observed between HADS anxiety score and diversion (r = −0.005, *p* = 0.95), reinterpretation (r = 0.01, *p* = 0.87) and self-encouragement (r = −0.04, *p* = 0.62) CSQ scores.

## 4. Discussion

This novel statistical approach based on finite mixture models allowed PSPS-T2 population clustering into two distinct classes depending on the effects of pain intensity, disability, and psychological distress on HRQoL. The first class, named the “disability class”, represented patients with HRQoL primarily impacted by functional disability and depression, while the HRQoL of the second class, named the “pain intensity class”, was more impacted by pain intensity and depression. Furthermore, level of education, perceived arduous working conditions, anxiety/depression, and coping strategies were significantly involved in the characterization of the disability and pain intensity classes.

### 4.1. A need for Multidimentional Composite Pain Assessment to Represent HRQoL Heterogeneity

Although the multidimensionality of chronic pain has been well-established [31,32,33] and reinforced by the biopsychosocial model [34,35] and the fear-avoidance model [36], pain management remains mainly focused on pain intensity relief. The vast majority of comparative pain research works are based on primary outcomes focused on pain reduction scores (VAS or NPRS). Regarding spinal cord stimulation, as an example, national and international guidelines recommend permanent implantation of neurostimulation devices based on a 30% or a 50% pain decrease during the trial period [37,38,39], but this can only reflect an angle of pain assessment prism [40,41]. Harmful consequences of focusing exclusively on pain intensity have been observed by Ballantyne and Sullivan [30], who contended that the multimodal pain management approaches proposed to chronic pain patients should include behavioral and physical-rehabilitation, which cannot be adequately evaluated using only pain intensity measures [41,42,43]. In a review [32], these authors claimed that it is not necessary to reduce pain intensity systematically in order to achieve adequate chronic pain management. This suggestion was supported by several studies indicating that multidisciplinary rehabilitation programs have shown greater effects on disability and quality of life than on pain intensity [44,45]. Furthermore, based on brain imaging data of 159 low back pain patients, Hashmi et al. [46] reported that pain intensity becomes less associated with nociception and more with emotional and psychological circuitry in chronic pain compared to acute pain.

Corroborating these findings, our results showed that psychological distress was significantly involved in the HRQoL of all the PSPS-T2 patients enrolled in this study. In addition to psychological distress, HRQoL was significantly impacted by functional disability for 68.7% of patients, and by pain intensity for 31.3% of the PSPS-T2 patients. All in all, our findings suggest that there are significant differences in the importance of sensory, functional, and emotional components in PSPS-T2 patients, which should be considered to propose specific care pathways according to a given patient and his or her intrinsic pain characteristics.

### 4.2. Characterisation of HRQoL Classes

The literature has shown that cultural and sociodemographic characteristics are related to how patients perceive their pain and to how much it impacts on their quality of life [47,48,49]. In a qualitative literature review including 77 articles on patients with general chronic pain, Samulowitz et al. [49] synthesized the gender biases often present in a patient–clinician relationship. The authors found that for men presenting with chronic pain, work and being a “breadwinner” were important and were linked to their sense of masculinity. The authors also found that physical activity, as an important part of male identity, was a recurring theme when men were describing their experience of living with pain. These findings confirm the hypothesis that functional capacity plays a significant role in male HRQoL patients, especially those with a physical job. In our study, we quantitatively found that the HRQoL of male patients who perceived their job as “physically demanding” was more impacted by functional disability than by pain intensity. For these patients, a deterioration in functional capacity generates a freeze in their professional activity and heavily impacts both self-image of the “breadwinner” and their financial status [47]. Although physical labor is more often associated with lower educational level, we found, in contrast, that the HRQoL of patients with a lower educational level was more impacted by pain intensity than functional disability. In this study, we did not find a significant relationship between educational level and how patients perceive their work (physical/not physical) (*p* = 0.71). Sociological studies clearly indicated, based on the traditional model of masculinity, particularly present in individuals with lower education levels and a lower social gradient of health (a concept describing the relationship between the socioeconomic status and health), that they traditionally have a more physically demanding work [35,50]. It could be thus hypothesized that PSPS-T2 patients with low educational level subjectively underestimate their work arduousness, while patients with a high educational level might overestimate the physical burden of their job, explaining the lack of association between education level and subjective job content. In addition, Roth and Geisser [48] have shown, in a study including 299 patients with chronic spinal pain, that patients with low educational levels had greater belief that pain is disabling and uncontrollable. Patients with a high educational level were more likely to participate in activities and to adopt more adaptive coping strategies [47]. Patients with higher education were more physically active and considered sports and physical activity as an active coping strategy. This indicates that for patients with a higher educational level, functional capacity preservation is necessary in order to cope with pain, which might explain why their HRQoL is more impacted by functional disability than by pain intensity. On the other hand, we cannot rule out a bidirectional effect, and it could be suggested that PSPS-T2 patients with a high level of education failing to cope appropriately with pain may have a decline in functional capacity. Furthermore, we found that the HRQoL of patients who frequently used adaptive coping strategies such as diverting attention from pain or reinterpreting pain were more affected by functional disability than by pain intensity. These results indicate that patients who use either emotion-based or active adaptive coping strategies are more impacted by functional disability. In a study of 103 patients with chronic PSPS-T2 pain [51], the authors found that physical activity practice was negatively associated with the use of maladaptive coping strategies such as catastrophizing. These findings suggest that a decrease of functional capacity might lead patients not to be able to cope with pain in a healthy manner, finally leading to deterioration in HRQoL. These results indicate that gender, work’s physical burden, level of education, and coping strategies are important factors that need to be considered during HRQoL assessment and in the process of identifying what is important for the patient.

### 4.3. Study Strengths and Limitations

Aside from the relatively large sample size and the prospective design, the strengths of this study also included the novel application of model-based statistical clustering. This allows overcoming the use of subjective goal identification tools. When patients are asked about what impacts them the most, the majority of patients tend to aim for pain intensity relief, as they believe that functional improvement is a necessary following of pain relief [52]. In our study, we found that the majority of patients’ quality of life is affected by disability more than pain perception. Statistical techniques allow for an identification of profiles based on the overall behavior and not the individual behavior, but they allow for an objective patient-profile identification, which is not achievable using self-reported tools and questionnaires.

Despite the study strengths, some limitations must be considered. First, our study sample was exclusively drawn from a PSPS-T2 population, which did not allow us to extend our findings to the general pain population, including acute and chronic general or widespread pain and non-operated patients. Another limitation is that we focused solely on pain intensity, functional disability and depression when describing HRQoL. Although it is clear that these three components represent major dimensions of quality of life, having significant consequences on patient HRQoL, there are other chronic pain components, which we deliberately did not include in our study, such as expectations, social insecurity or lack of sleep, which might also contribute to building on a holistic HRQoL model. A new cohort study including patients with widespread acute and chronic pain, aiming to focus with more clarity on all dimensions of quality of life could yield more specific biopsychosocial clustering and optimized characterization of pain-related quality of life. Furthermore, we assume that the extension of this pilot work should be based on a larger cohort, as a prospective registry, to bridge this gap and address two specific goals—external validation and expansion of the results of this study—since there is an obvious lack in literature regarding how chronic pain patients belonging to distinct latent subpopulations are affected individually by different components of pain.

### 4.4. Therapeutical Implications

From a clinical perspective, the identification of patient clusters, determined from their social and cognitive-behavioral characteristics, could help physicians to tailor personalized care, to propose “à la carte” programs and ultimately reinforce therapeutic alliance, thereby improving patient outcomes. Patients highly impacted by their functional disability could be preferentially oriented towards therapies and programs that have demonstrated a specific impact on disability, such as cognitive-behavioral-based physical therapies [53,54], to cope with their body; conversely, patients predominantly impacted by pain intensity could be oriented towards specific pain relief therapies to cope with pain perception, emotions and revisit their mind. It appears clear, from the extreme variability of treatments available to patients presenting with chronic pain, that each treatment strategy has a preferential impact on pain intensity or on functional disability [7]. Given these findings, we should probably insist on patient profiling to redesign personalized pain patient care pathways and to put the patient back at the center of the pain puzzle.

## 5. Conclusions

Applying a mixture of mixed effects models to data of chronic pain patients might lead to the development of new pain evaluation strategies and to better understanding of HRQoL dimensions. The results of this study can be considered as a starting point to refine a multidimensional, personalized, cluster-based, composite evaluation of HRQoL in chronic pain patients, which could impact on the optimization of pain management.

## Figures and Tables

**Table 1:** Patients baseline sociodemographic and clinical characteristics (*n* = 198).

Variables	*n*	%
Mean age (SD)	53 (13)	
Gender		
Male	87	43.9
Female	111	56.1
Professional status		
In professional activity	41	20.7
Retired	38	19.2
Disability	38	19.2
Sick leave	38	19.2
Long-term illness	16	8.1
Unemployment	7	3.5
Without professional activity	20	10.1
Educational level		
≤12 years	153/193	79.3
>12 years	40/193	20.7
Number of spinal surgeries		
2	99	50.0
3	59	29.8
4	28	14.1
5	8	4.0
6+	4	2.0
Neuropathic pain (DN4 * score ≥ 4)		
Yes	147/184	80
No	37/184	20
Pain duration		
≤5 years	46/198	23
>5 years	152/198	77

* DN4: Douleur Neuropathique 4Questions, a 10-item questionnaire with a score ranging from 0 to 10.

**Table 2:** Correlations between EQ-5D index, ODI, HADS depression score and CSQ catastrophizing at baseline and at 12-month follow-up.

	Baseline			12-Month Follow-Up		
Variables	ODI	NPRS	HADS Dep ^T^	ODI	NPRS	HADS Dep ^T^
EQ-5D	−0.66 ***	−0.35 ***	−0.44 ***	−0.77 ***	−0.55 ***	−0.56 ***
ODI	-	0.44 ***	0.40 ***	-	0.54 ***	0.57 ***
NPRS	-	-	0.33 ***	-	-	0.39 ***

EQ-5D: EuroQol-5 Dimensions, NPRS: Numeric Pain Rating Scale, ODI: Oswestry Disability Index, HADS: Hospital Anxiety and Depression Scale, CSQ: Coping Strategies Questionnaire. ^T^ HADS depression subscale. * *p* < 0.01, ** *p* < 0.001, *** *p* < 0.0001.

**Table 3:** Global, class 1 and class 2 mixed effects models representing the effects of functional disability, pain intensity and depression on health-related quality of life.

	Standard 1-Class Mixed Effects Model			Results of the Two-Class Mixture Model					
				Class 1 Model			Class 2 Model		
Variables	Standardized Coefficient	Standard Error	*p*-Value	Standardized Coefficient	Standard Error	*p*-Value	Standardized Coefficient	Standard Error	*p*-Value
Intercept	−0.0029	0.037	0.93	−0.0060	0.037	0.94	-	-	-
ODI (%)	−0.48	0.034	<0.0001	−0.76	0.074	<0.0001	−0.11	0.095	0.23
NPRS	−0.13	0.027	<0.0001	0.039	0.041	0.46	−0.35	0.065	<0.0001
HADS depression	−0.20	0.030	<0.0001	−0.19	0.044	<0.0001	−0.22	0.057	0.0001

NPRS: Numeric Pain Rating Scale, ODI: Oswestry Disability Index, HADS: Hospital Anxiety and Depression Scale.

**Table 4:** Demographic and cognitive-behavioral factors associated with class membership.

Variable	Mean (sd)/*n*(%)		Standardized Coefficients	95% Confidence Interval	Adjusted *p*-Value
	Disability Class (*n* = 136)	Pain Intensity Class (*n* = 62)			
Intercept	-	-	−1.26	[−1.79, −0.73]	<0.0001
Age (years)	52.01 (12.08)	54.47 (13.41)	0.28	[−0.28, 0.84]	0.31
Gender (male)	60/136 (44%)	27/62 (44%)	−0.24	[−0.73, 0.25]	0.32
Level of study (years)	11.36 (3.14)	9.47 (4.36)	−0.61	[−1.15, −0.08]	0.020
Perceived physical job					
Working in a job perceived as physical	17 (13%)	9 (15%)	0.08	[−0.46, 0.63]	0.77
Working in a job not perceived as physical	10 (7%)	5 (8%)	0.04	[−0.48, 0.57]	0.87
Not in professional activity	109 (80%)	48 (77%)	−	-	
Global NPRS at baseline	5.91 (1.41)	6.47 (1.57)	0.65	[0.05, 1.24]	0.029
ODI percentage at baseline	33.92 (10.83)	34.98 (13.40)	−0.23	[−0.86, 0.39]	0.45
HADS depression subscale	8.44 (3.88)	9.02 (3.83)	0.88	[0.17, 1.59]	0.012
HADS anxiety scale	10.43 (3.79)	9.03 (3.83)	−0.98	[−1.70, −0.27]	0.005
CSQ catastrophizing scale	14.17 (4.64)	12.98 (4.18)	−0.60	[−1.28, 0.07]	0.07
CSQ diversion	12.68 (3.50)	11.30 (3.72)	−0.44	[−0.97, 0.08]	0.09
CSQ pain ignorance	9.37 (3.23)	9.27 (3.64)	−0.44	[−0.96, 0.08]	0.13
CSQ pain reinterpretation	6.03 (2.55)	5.09 (1.71)	−0.51	[−1.03, 0.02]	0.12
CSQ self-encouragement	10.61 (2.78)	10.08 (2.63)	0.81	[0.29, 1.34]	0.011
Pain duration (years)	4.43 (5.92)	4.64 (6.35)	−0.20	[−0.73, 0.32]	0.37
Gender (male) x physical job *					
Male working in a physical job	12/60 (20%)	2/27 (7%)	−0.55	[−1.08, −0.03]	0.027
Male working but not in a physical job	3/60 (5%)	2/27 (7%)	−0.17	[−0.69, 0.36]	0.53
Male and not in professional activity	45/60 (75%)	23/27 (86%)	-	-	-

* The interaction term between the “Gender” and the “physical job” variables. NPRS: Numeric Pain Rating Scale, ODI: Oswestry Disability Index, CSQ: Coping Strategies Questionnaire, HADS: Hospital Anxiety and Depression Scale.

## Data Availability

Not applicable.

## References

[1] Christelis N., Simpson B., Russo M., Stanton-Hicks M., Barolat G., Thomson S., Schug S., Baron R., Buchser E., Carr D.B. (2021). Persistent Spinal Pain Syndrome: A Proposal for Failed Back Surgery Syndrome and ICD-11. Pain Med..

[2] Rigoard P., Gatzinsky K., Deneuville J.-P., Duyvendak W., Naiditch N., Van Buyten J.-P., Eldabe S. (2019). Optimizing the Management and Outcomes of Failed Back Surgery Syndrome: A Consensus Statement on Definition and Outlines for Patient Assessment. Pain Res. Manag..

[3] Scalone L., Zucco F., Lavano A., Costantini A., Rose M.D., Poli P., Fortini G., Demartini L., Simone E.D., Menardo V. (2018). Benefits in Pain Perception, Ability Function and Health-Related Quality of Life in Patients with Failed Back Surgery Syndrome Undergoing Spinal Cord Stimulation in a Clinical Practice Setting. Health Qual. Life Outcomes.

[4] Goudman L., Smedt A.D., Putman K., Moens M. (2020). Long-Term Quality of Life and Work Status after High-Dose Spinal Cord Stimulation in Patients with Failed Back Surgery Syndrome: A Secondary Analysis of Real-World Data. J. Neurosurg. Spine.

[5] De Jaeger M., Goudman L., Putman K., De Smedt A., Rigoard P., Geens W., Moens M. (2020). The Added Value of High Dose Spinal Cord Stimulation in Patients with Failed Back Surgery Syndrome after Conversion from Standard Spinal Cord Stimulation. J. Clin. Med..

[6] Castelli L., Tesio V., Colonna F., Molinaro S., Leombruni P., Bruzzone M., Fusaro E., Sarzi-Puttini P., Torta R. (2012). Alexithymia and Psychological Distress in Fibromyalgia: Prevalence and Relation with Quality of Life. Clin. Exp. Rheumatol..

[7] Davies M., Brophy S., Williams R., Taylor A. (2006). The Prevalence, Severity, and Impact of Painful Diabetic Peripheral Neuropathy in Type 2 Diabetes. Diabetes Care.

[8] Hasenbring M.I., Plaas H., Fischbein B., Willburger R. (2006). The Relationship between Activity and Pain in Patients 6 Months after Lumbar Disc Surgery: Do Pain-Related Coping Modes Act as Moderator Variables?. Eur. J. Pain.

[9] Langley P., Pérez Hernández C., Margarit Ferri C., Ruiz Hidalgo D., Lubián López M. (2011). Pain, Health Related Quality of Life and Healthcare Resource Utilization in Spain. J. Med. Econ..

[10] Kovacs F.M., Abraira V., Zamora J., Teresa Gil del Real M., Llobera J., Fernández C., Bauza J.R., Bauza K., Coll J., Cuadri M. (2004). Correlation between Pain, Disability, and Quality of Life in Patients with Common Low Back Pain. Spine.

[11] Rapti E., Damigos D., Apostolara P., Roka V., Tzavara C., Lionis C. (2019). Patients with Chronic Pain: Evaluating Depression and Their Quality of Life in a Single Center Study in Greece. BMC Psychol..

[12] Proust-Lima C., Philipps V., Liquet B. (2017). Estimation of Extended Mixed Models Using Latent Classes and Latent Processes: The R Package Lcmm. J. Stat. Softw..

[13] Chen Y., Campbell P., Strauss V.Y., Foster N.E., Jordan K.P., Dunn K.M. (2018). Trajectories and Predictors of the Long-Term Course of Low Back Pain: Cohort Study with 5-Year Follow-Up. Pain.

[14] Dunn K.M., Jordan K., Croft P.R. (2006). Characterizing the Course of Low Back Pain: A Latent Class Analysis. Am. J. Epidemiol..

[15] Herdman M., Gudex C., Lloyd A., Janssen M., Kind P., Parkin D., Bonsel G., Badia X. (2011). Development and Preliminary Testing of the New Five-Level Version of EQ-5D (EQ-5D-5L). Qual. Life Res..

[16] Obradovic M., Lal A., Liedgens H. (2013). Validity and Responsiveness of EuroQol-5 Dimension (EQ-5D) versus Short Form-6 Dimension (SF-6D) Questionnaire in Chronic Pain. Health Qual. Life Outcomes.

[17] Tran B.X., Ohinmaa A., Nguyen L.T. (2012). Quality of Life Profile and Psychometric Properties of the EQ-5D-5L in HIV/AIDS Patients. Health Qual. Life Outcomes.

[18] Vartiainen P., Mäntyselkä P., Heiskanen T., Hagelberg N., Mustola S., Forssell H., Kautiainen H., Kalso E. (2017). Validation of EQ-5D and 15D in the Assessment of Health-Related Quality of Life in Chronic Pain. Pain.

[19] Ferreira-Valente M.A., Pais-Ribeiro J.L., Jensen M.P. (2011). Validity of Four Pain Intensity Rating Scales. Pain.

[20] Fairbank J.C., Couper J., Davies J.B., O’Brien J.P. (1980). The Oswestry Low Back Pain Disability Questionnaire. Physiotherapy.

[21] Lee C.-P., Fu T.-S., Liu C.-Y., Hung C.-I. (2017). Psychometric Evaluation of the Oswestry Disability Index in Patients with Chronic Low Back Pain: Factor and Mokken Analyses. Health Qual. Life Outcomes.

[22] Vianin M. (2008). Psychometric Properties and Clinical Usefulness of the Oswestry Disability Index. J. Chiropr. Med..

[23] Zigmond A.S., Snaith R.P. (1983). The Hospital Anxiety and Depression Scale. Acta Psychiatr. Scand..

[24] Bjelland I., Dahl A.A., Haug T.T., Neckelmann D. (2002). The Validity of the Hospital Anxiety and Depression Scale. An Updated Literature Review. J. Psychosom. Res..

[25] Turk D.C., Dworkin R.H., Trudeau J.J., Benson C., Biondi D.M., Katz N.P., Kim M. (2015). Validation of the Hospital Anxiety and Depression Scale in Patients with Acute Low Back Pain. J. Pain.

[26] Sagheer M.A., Khan M.F., Sharif S. (2013). Association between Chronic Low Back Pain, Anxiety and Depression in Patients at a Tertiary Care Centre. J. Pak. Med. Assoc..

[27] Irachabal S., Koleck M., Rascle N., Bruchon-Schweitzer M. (2008). Pain coping strategies: French adaptation of the coping strategies questionnaire (CSQ-F). Encephale.

[28] Efron B. (1979). Bootstrap Methods: Another Look at the Jackknife. Ann. Stat..

[29] Proust C., Jacqmin-Gadda H. (2005). Estimation of Linear Mixed Models with a Mixture of Distribution for the Random Effects. Comput. Methods Programs Biomed..

[30] Schwarz G. (1978). Estimating the Dimension of a Model. Ann. Stat..

[31] Ballantyne J.C., Sullivan M.D. (2015). Intensity of Chronic Pain--The Wrong Metric?. N. Engl. J. Med..

[32] Sullivan M.D., Ballantyne J.C. (2016). Must We Reduce Pain Intensity to Treat Chronic Pain?. Pain.

[33] Dorfman D., George M.C., Robinson-Papp J., Rahman T., Tamler R., Simpson D.M. (2016). Patient Reported Outcome Measures of Pain Intensity: Do They Tell Us What We Need to Know?. Scand. J. Pain.

[34] Gatchel R.J., Peng Y.B., Peters M.L., Fuchs P.N., Turk D.C. (2007). The Biopsychosocial Approach to Chronic Pain: Scientific Advances and Future Directions. Psychol. Bull..

[35] Naiditch N., Billot M., Moens M., Goudman L., Cornet P., Le Breton D., Roulaud M., Ounajim A., Page P., Lorgeoux B. (2021). Persistent Spinal Pain Syndrome Type 2 (PSPS-T2), a Social Pain? Advocacy for a Social Gradient of Health Approach to Chronic Pain. J. Clin. Med..

[36] Crombez G., Eccleston C., Van Damme S., Vlaeyen J.W.S., Karoly P. (2012). Fear-Avoidance Model of Chronic Pain: The Next Generation. Clin. J. Pain.

[37] National Institute for Health and Clinical Excellence Spinal Cord Stimulation for Chronic Pain of Neuropathic or Ischaemic Origin. https://www.nice.org.uk/guidance/ta159.

[38] Rigoard P., Billot M., Ingrand P., Durand-Zaleski I., Roulaud M., Peruzzi P., Hieu P.D., Voirin J., Raoul S., Page P. (2021). How Should we Use Multicolumn Spinal Cord Stimulation to Optimize Back Pain Spatial Neural Targeting? A Prospective, Multicenter, Randomized, Double-Blind, Controlled Trial ( ESTIMET Study). Neuromodulation Technol. Neural Interface.

[39] Centers for Medicare & Medicaid Services National Coverage Determination (NCD) for Electrical Nerve Stimulators. https://www.cms.gov/medicare-coverage-database/view/ncd.aspx?NCDId=240.

[40] Jaeger M.D., Goudman L., Eldabe S., Dongen R.V., Smedt A.D., Moens M. (2021). The Association between Pain Intensity and Disability in Patients with Failed Back Surgery Syndrome, Treated with Spinal Cord Stimulation. Disabil. Rehabil..

[41] Rigoard P., Ounajim A., Goudman L., Louis P.-Y., Yousri S., Roulaud M., Bouche B., Wood C., Page P., Lorgeoux B. (2021). A Novel Multi-Dimensional Clinical Response Index Dedicated to Improve Pain Global Assessment in Patients with Persistent Spinal Pain Syndrome after Spinal Surgery, Based on a Real-Life Prospective Multicentric Study (PREDIBACK) and Machine Learning Techniques. Preprints.

[42] Goudman L., Moens M. (2020). Moving Beyond a Pain Intensity Reporting: The Value of Goal Identification in Neuromodulation. Neuromodulation.

[43] Goudman L., De Smedt A., Eldabe S., Rigoard P., Linderoth B., De Jaeger M., Moens M., Consortium D. (2020). High-Dose Spinal Cord Stimulation for Patients with Failed Back Surgery Syndrome: A Multicenter Effectiveness and Prediction Study. Pain.

[44] Kamper S.J., Apeldoorn A.T., Chiarotto A., Smeets R.J.E.M., Ostelo R.W.J.G., Guzman J., van Tulder M.W. (2014). Multidisciplinary Biopsychosocial Rehabilitation for Chronic Low Back Pain. Cochrane Database Syst. Rev..

[45] Marris D., Theophanous K., Cabezon P., Dunlap Z., Donaldson M. (2021). The Impact of Combining Pain Education Strategies with Physical Therapy Interventions for Patients with Chronic Pain: A Systematic Review and Meta-Analysis of Randomized Controlled Trials. Physiother. Theory Pract..

[46] Hashmi J.A., Baliki M.N., Huang L., Baria A.T., Torbey S., Hermann K.M., Schnitzer T.J., Apkarian A.V. (2013). Shape Shifting Pain: Chronification of Back Pain Shifts Brain Representation from Nociceptive to Emotional Circuits. Brain.

[47] Leclerc A., Gourmelen J., Chastang J.-F., Plouvier S., Niedhammer I., Lanoë J.-L. (2009). Level of Education and Back Pain in France: The Role of Demographic, Lifestyle and Physical Work Factors. Int. Arch. Occup. Environ. Health.

[48] Roth R.S., Geisser M.E. (2002). Educational Achievement and Chronic Pain Disability: Mediating Role of Pain-Related Cognitions. Clin. J. Pain.

[49] Samulowitz A., Gremyr I., Eriksson E., Hensing G. (2018). “Brave Men” and “Emotional Women”: A Theory-Guided Literature Review on Gender Bias in Health Care and Gendered Norms towards Patients with Chronic Pain. Pain Res. Manag..

[50] Naiditch N., Billot M., Goudman L., Cornet P., Roulaud M., Ounajim A., Page P., Lorgeoux B., Baron S., Nivole K. (2021). Professional Status of Persistant Spinal Pain Syndrome Patients after Spinal Surgery (PSPS-T2): What Does Really Matter? A pro-Spective Study Introducing the Concept of “Adapted Profession-al Activity” Infering from Clinical, Psychological and Social influence. J. Clin. Med..

[51] Higuchi D. (2020). Adaptive and Maladaptive Coping Strategies in Older Adults with Chronic Pain after Lumbar Surgery. Int. J. Rehabil. Res..

[52] Henry S.G., Bell R.A., Fenton J.J., Kravitz R.L. (2017). Goals of Chronic Pain Management: Do Patients and Primary Care Physicians Agree and Does It Matter?. Clin. J. Pain.

[53] Archer K.R., Devin C.J., Vanston S.W., Koyama T., Phillips S.E., Mathis S.L., George S.Z., McGirt M.J., Spengler D.M., Aaronson O.S. (2016). Cognitive-Behavioral-Based Physical Therapy for Patients with Chronic Pain Undergoing Lumbar Spine Surgery: A Randomized Controlled Trial. J. Pain.

[54] Barnhoorn K.J., van de Meent H., van Dongen R.T.M., Klomp F.P., Groenewoud H., Samwel H., Nijhuis-van der Sanden M.W.G., Frölke J.P.M., Staal J.B. (2015). Pain Exposure Physical Therapy (PEPT) Compared to Conventional Treatment in Complex Regional Pain Syndrome Type 1: A Randomised Controlled Trial. BMJ Open.

